# Counteracting Data Bias and Class Imbalance—Towards a Useful and Reliable Retinal Disease Recognition System

**DOI:** 10.3390/diagnostics13111904

**Published:** 2023-05-29

**Authors:** Adam R. Chłopowiec, Konrad Karanowski, Tomasz Skrzypczak, Mateusz Grzesiuk, Adrian B. Chłopowiec, Martin Tabakov

**Affiliations:** 1 Department of Artificial Intelligence, Wroclaw University of Science and Technology, Wybrzeże Wyspianskiego 27, 50-370 Wroclaw, Poland; 254518@student.pwr.edu.pl (A.R.C.); 254529@student.pwr.edu.pl (M.G.); 254517@student.pwr.edu.pl (A.B.C.); martin.tabakow@pwr.edu.pl (M.T.); 2 Faculty of Medicine, Wroclaw Medical University, Wybrzeże Ludwika Pasteura 1, 50-367 Wroclaw, Poland; t.skrzypczak.pl@gmail.com

**Keywords:** deep learning, medical image classification, convolutional neural networks

## Abstract

Multiple studies presented satisfactory performances for the treatment of various ocular diseases. To date, there has been no study that describes a multiclass model, medically accurate, and trained on large diverse dataset. No study has addressed a class imbalance problem in one giant dataset originating from multiple large diverse eye fundus image collections. To ensure a real-life clinical environment and mitigate the problem of biased medical image data, 22 publicly available datasets were merged. To secure medical validity only Diabetic Retinopathy (DR), Age-Related Macular Degeneration (AMD) and Glaucoma (GL) were included. The state-of-the-art models ConvNext, RegNet and ResNet were utilized. In the resulting dataset, there were 86,415 normal, 3787 GL, 632 AMD and 34,379 DR fundus images. ConvNextTiny achieved the best results in terms of recognizing most of the examined eye diseases with the most metrics. The overall accuracy was 80.46 ± 1.48. Specific accuracy values were: 80.01 ± 1.10 for normal eye fundus, 97.20 ± 0.66 for GL, 98.14 ± 0.31 for AMD, 80.66 ± 1.27 for DR. A suitable screening model for the most prevalent retinal diseases in ageing societies was designed. The model was developed on a diverse, combined large dataset which made the obtained results less biased and more generalizable.

## 1. Introduction

According to the first World Report on Vision issued by the World Health Organization (WHO) in 2019, approximately 2.2 billion people had vision impairment or blindness, globally [1]. This number is expected to rise because of the growth of the global population and the changes in its age structure [2]. The soaring work effort associated with the ageing population is an overwhelming problem for the limited number of eye care providers [3,4]. Efficiency and effectiveness enhancements should be a fundamental response to a projected undersupply of eye care providers [4].

Recent research has proved that deep learning systems could be useful in delivering patient care in a real-world setting [5]. Multiple satisfactory performances of artificial intelligence models for the automated detection of ocular diseases were reported [5,6,7,8,9]. Clinically useful models should differentiate the most distressing diseases: diabetic retinopathy (DR), glaucoma (GL) and age-related macular degeneration (AMD) [2,10] from a healthy fundus, with high sensitivity and specificity. These diseases are prevalent in ageing populations, which makes them suitable targets for a screening system [1,2,10]. Recently, there have been several multiclass models published that at least partially meet these conditions [11,12,13,14,15,16,17,18]. However, all these models had multiple limitations.

Most of the published multiclass models were developed on a single dataset [11,13,15,16,17], mainly the Ocular Disease Intelligent Recognition (ODIR) database [13,15,17,19]. This could lead to a potential bias in the development of machine learning models. A single database is often a survey of a certain population, collected with a small number of cameras in several medical centers by a limited number of investigators. Data gathered in similar environments, or a single process, may not apply to other clinics due to different cameras, ethnicity, or an image acquisition technique. These models are not generalizable to the overall patient population. One of the most effective strategies to mitigate these biases is to compile a large-scale, multiethnic dataset that would be representative and would simulate a real-world environment for model training [20]. The collection of such a dataset would contribute to better accuracy and fairness in the decision-making process. Such an approach was partially adopted by previous works [12,14] although the clarity of data selection and quantity of merged datasets could still be improved.

Class imbalanced datasets occur in many real-world applications where class distributions of data are highly imbalanced [21]. Many classification learning algorithms have lower predictive accuracy for infrequent classes [21]. Models misclassify diseases with lower prevalence in the retinal images database. Merging multiple different datasets could even potentiate this issue. Due to this imbalance, the accuracy of detection or classification of disease is relatively low [15]. Most of the published studies [11,12,14,16,17] did not address the problem, which could influence the results. Common techniques for handling class imbalance problems involve reweighting, resampling and other algorithmic solutions [22,23]. Applying them to a large dataset could help in the recognition of less prevalent diseases.

Three out of the eight most recently published works [9,11,14] utilized private datasets. These are often formally available upon correspondence with a reasonable request. Potentially, these data could never be made available to the public. Research transparency could be put under question, as these studies may not be reproducible due to data unavailability.

Almost all published models included cataracts as a retinal disease [11,12,13,14,15,17,18]. A cataract is a cloudification of the natural intraocular lens and is not classified as a retinal disorder by the medical literature [24] In a cataract, the fundus image is not visible or is heavily distorted when photographed [24]. It seems reasonable to assume that the usage of such images in multiclass model development aimed at retinal diseases has influenced the results and has no utility in the screening and diagnostic process.

Although the assessment of the retinal fundus in both myopia and hypertensive retinopathy may have some usefulness in routine patient screening, no medical guidelines recommend this in clinical practice. Inclusion of these diseases in multiclass models developed by multiple previous investigators [11,13,14,15] could lead to unnecessary class proliferation, influence results and lead to lower screening utility. Similarly, the inclusion of relatively rare diseases like retinitis pigmentosa had a limited purpose in model development. We assumed that the perfect screening multiclass model should be focused on the most common retinal diseases that distress whole nations.

The primary aim of this study was to create an image recognition model for retinal disease screening in ageing, developed countries. The model was developed on one cumulative dataset and differentiated DR, AMD and GL from a normal eye fundus for the best clinical utility. The created database utilized multiple types of fundus cameras, evaluated by various retinal experts, and represents multiple nationalities, which approximates to the true real-world environment. This results in mitigation of the data bias problem. The utilized data had clear selection criteria and usage of only publicly available datasets made our experiment reproducible. The secondary aim was to address the problem of class imbalance which is a result of merging multiple different large datasets. To achieve that we proposed to combine transfer learning, loss function weighting and two-stage learning techniques.

## 2. Materials and Methods

To ensure our database minimizes the problem of biased medical image data, we collected and merged 22 publicly available fundus datasets containing images of any of the diseases classified in our paper. We selected only the data strictly related to the diagnostic process of the pathologies considered. Such a dataset consists of fundus images obtained from multiple hospitals and clinical centers all around the world, providing data from various ethnic and demographic groups. Such data contain noise, overexposure, underexposure and other visual artifacts, as well as perfectly prepared fundus images. Similar artifacts may be commonly encountered in hospitals due to human or hardware errors. The images were taken with various cameras, mydriatic and non-mydriatic. Such a wide range of images provides the least biased and most real-world adjusted clinical usage database that has been collected in the studies up to the present, consisting only of public data, which has been properly filtered for selected pathologies and their diagnostic process. Therefore, unlike studies using single public or private datasets, which are possibly biased, we provide the most reliable results for the task of classification of fundus diseases.

**Figure 1:**
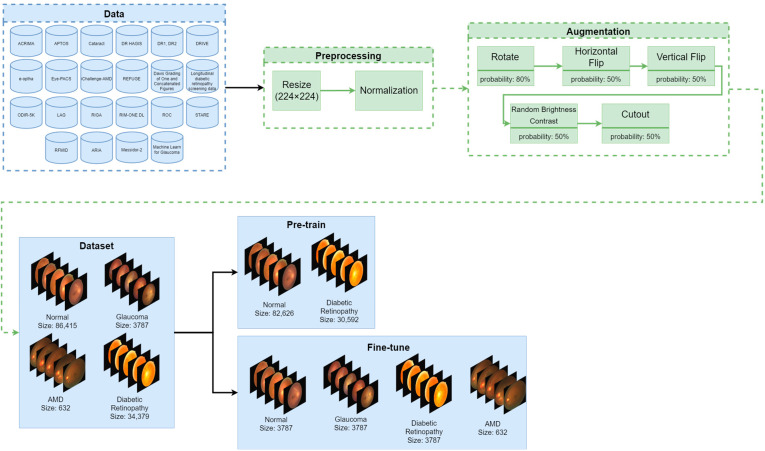
Data processing pipeline. We gathered 22 databases and merged them into one large dataset. We resized all images to 224 × 224 and normalized them by applying mean and std values derived from ImageNet-1K. Further, we split the data into two subgroups—the one used in pre-training and the other used in fine-tuning. During the training process, we dynamically augmented the images with fixed probabilities.

**Figure 2:**
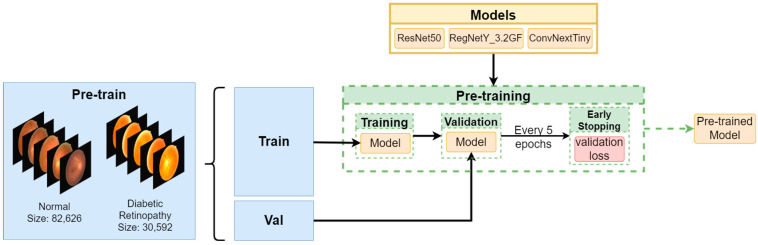
Pre-training flow. We split the pre-training data into parts: train and validation set. We pre-train each model, performing evaluation every epoch while monitoring validation loss and applying early stopping with patients of five epochs.

**Figure 3:**
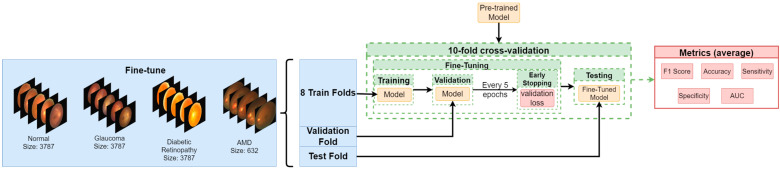
Fine-tuning workflow. We performed 10-fold cross-validation, selecting one-fold for a validation set and one-fold for a test set at each cross-validation step. We fine-tuned each model on a training set while monitoring validation loss and applying early stopping with patients of five epochs. In each cross-validation step, each fine-tuned model was evaluated on a test set, providing a set of metrics for that step. These performance metrics were further averaged.

In our experiments, we had to tackle important problems related to medical image classification in general. We used state-of-the-art models (ConvNext [25] RegNet [26] ResNet [27]) employed in computer vision and verified their accuracy on biomedical data. Further, we present data augmentation methods used to avoid overfitting which is a common problem in the domain [28,29,30]. To address the problem of class imbalance we split the dataset into two parts: pre-training and fine-tuning. Splitting the data into train, validation and test sets is described in the section Fine-Tuning. Our study workflow is presented in Figure 1, Figure 2 and Figure 3.

### 2.1. Models

We chose Convolutional Neural Networks (CNNs) for fundus image classification as widely used and well-performing models in image recognition tasks. CNNs consist of two main parts:

- A feature extractor built mostly with convolutional layers, used to capture increasingly abstract image features, which are then compressed into a vector, called feature-embedding, during the process.
- A classifier containing mainly dense, fully connected layers, responsible for the classification of a feature-embedding vector.

In our experiments we decided to use recently published state-of-the-art models of CNNs: ConvNext [25] and RegNet [26] and compare their performance to the most-used architecture in image classification tasks—ResNet [13,27,31,32]. ConvNext architecture was inspired by Hierarchical Transformers [33]. It modernizes ResNet by employing various macro and micro design choices from Transformers and other popular CNNs like ResNext [34] or MobileNetV2 [35]. RegNet architectures are a family of CNNs that come from the progressively designed RegNet design space. They have proved to be effective in many computational regimes [26,36]. Current state-of-the-art architectures are understudied in the biomedical domain, although recent studies prove their potential in diverse applications [36,37,38,39,40]. Therefore, we found it valuable to verify their superiority over commonly used architectures in medical image classification. The most widely used architecture of ResNet is ResNet50, which we found suitable for the data we collected. To match its size, we chose ConvNextTiny and RegNetY3_2gf. All architectures were imported from the torchvision package [41].

### 2.2. Data Augmentation

Lack of data is a common problem for applications of deep learning techniques in the biomedical domain [42,43,44,45]. Therefore, we decided to use data augmentation, with a library provided by Buslaev et al. [46], to cover a larger space of possible inputs to our networks to increase robustness. Fundus images in real-world cases are transformed affinely—images are often rotated or inverted. Moreover, a natural characteristic of medical images is underexposure or overexposure due to hardware or human mistakes [47]. Images from different databases come with a range of resolutions, so there was a need to standardize their size. Taking such features into consideration, we decided to use the transformations described in Table 1. We have additionally used cutouts for regularization [48]. No data augmentation was used during the validation or testing phase.

### 2.3. Model Training

Data imbalance is a common problem in medical image classification. Naturally, some diseases are rare and difficult to classify, or data are collected from limited sources due to data collection costs or law-related issues. In such cases, data imbalance occurs. The problem is potentiated when compiling a large and diverse dataset from many smaller datasets. We proposed to use transfer learning and two-stage learning to better adjust our models to fundus images classification task. A two-stage learning procedure consists of pre-training a model on excess domain data and fine-tuning on thresholded data. The procedure described is similar to the two-phase learning reported by Johnson et al. [23]. Although it differs in the way it defines both stages, two-phase learning first pre-trains a model with thresholded data and then fine-tunes it using all data. For the pre-training part, we selected an excessive amount of normal and diabetic retinopathy images over the threshold of the cardinality of glaucoma images. The fine-tuning part consisted of the remaining normal, diabetic retinopathy, AMD, and glaucoma images. The summary of the data split is presented in Table 2. Such data division allows us to adjust a model to the domain problem with excess data from the major classes, reducing general overfitting to them during fine-tuning, by matching their cardinality with minor classes. The pre-training dataset was used in the pre-training phase and the fine-tuning dataset was used in the fine-tuning phase. We used Weights & Biases [49] for experiment tracking and visualizations to develop insights for this paper.

### 2.4. Pre-Training

In the pre-training phase, we used ImageNet-1K pre-trained models. We removed the fully-connected layer and replaced it with a new, randomly initialized one which had only two outputs—for diabetic retinopathy and normal image predictions. We froze half of the CNN layers to use the pre-trained feature-extraction abilities. Next, we trained each model with early stopping with patients of 5 epochs, monitoring validation-set loss. For the optimizer we chose Radam [50] with a learning rate 3^−4^, batch size 32 and weight decay of 1^−5^. To further tackle the problem of class imbalance we decided to use weighted cross entropy loss with weights 1 and 2 for Normal and Diabetic Retinopathy classes, respectively. We used a cosine-annealing learning rate scheduler [51], with *Tmax* = 20, *ηmin* = 10^−5^ and *ηmax* = 3 × 10^−4^.

### 2.5. Fine Tuning

From the model obtained in the fine-tuning phase, we removed the fully connected layer and replaced it with a new, randomly initialized one which had four outputs, unlike in pre-training. Similarly, we froze half of the convolutional layers of the model. To perform an unbiased evaluation, we trained our models in a 10-fold cross-validation process. We trained each model 10 times, every time choosing a different part of the dataset for the test set, another for the validation set and the rest for the train set. The experiments were performed with the same hyperparameters as in the pre-training phase, except for the weights used in cross-entropy—here they are equal to 1, 0.9, 1.5, 1.2 for normal, glaucoma, AMD, and diabetic retinopathy classes, respectively. We report the average results of all runs for each model.

### 2.6. Verification of Other Resampling Methods

Resampling methods are widely used in the literature on class imbalance [23,52,53]. To present a fair comparison and verify the results provided on data with mitigated bias we performed experiments using other resampling methods, namely: random minority oversampling (ROS) and random majority undersampling (RUS). Similarly to the procedure described in the Fine Tuning section, we trained models in a 10-fold cross-validation process. To maintain comparability with the outcomes of two-stage learning and align the class ratios for validation and test sets with our Fine Tuning phase, we applied a threshold during each cross-validation iteration for validation and test folds. This threshold ensured that the number of normal and diabetic retinopathy images matched the cardinality of glaucoma images. We used the same hyperparameters as in our Fine Tuning phase. All experiments were performed using the ConvNextTiny architecture pretrained on ImageNet-1K.

## 3. Results

### 3.1. Dataset

In the resulting dataset, there are 86,415 normal, 3787 glaucoma, 632 AMD and 34,379 diabetic retinopathy fundus images. The summary and medical characteristics of the datasets are presented in Table 3.

Most datasets were annotated by experts, except four for which the data acquisition process was not described: APTOS 2019 Blindness Detection Dataset [55], Cataract [56], Machine learning for glaucoma [60] and BAIDU: iChallenge-AMD [62].

### 3.2. Evaluation Criteria

In our experiments, to leverage the advantage of a diverse real-world dataset we report mean and standard deviation over 10 runs in a 10-fold cross-validation process, therefore ensuring that every part of the dataset was used for evaluation. We used 5 metrics for every class: Accuracy, F1-Score, Sensitivity, Specificity, and AUC, and then we also averaged them across classes and reported the overall accuracy. For class-specific metrics, we used the one-versus-rest technique. Such a wide set of metrics allows a thorough examination of the models’ performance with respect to every disease [73,74].

### 3.3. Performance

In Table 4 we present the results of our experiments. ConvNextTiny achieved the best results in terms of recognizing most of the eye diseases examined with the most metrics.

**Figure 4:**
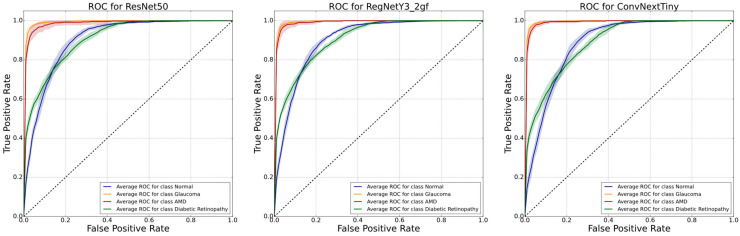
Performance of our models shown with ROC plots. Each disease average-classification metric is shown with a different color. The area surrounding each line represents a standard deviation of ROC.

It specifically excels over ResNet50 in the F1-Score for AMD with a difference of 1.2 pp. This proves the purposefulness of choosing modern state-of-the-art architectures for medical experiments. The ResNet50 model achieved the best results at recognizing glaucoma. RegNetY3_2gf scored the worst results at recognizing every disease with respect to the most metrics. Figure 4 summarizes the performance of each model with ROC curves for all diseases with their respective standard deviation. These curves show similar trends for all diseases across all models. ConvNextTiny achieved higher results than ResNet50 with an AUC of 90.64 and 91.65 for normal and diabetic retinopathy images, respectively.

### 3.4. Comparison of Resampling Methods

The results of experiments with other resampling methods are presented in Table 5. As in the previous experiments, we report mean and std over the cross-validation process. ROS performed the best with respect to most metrics. Most notably it achieved a difference of 0.7 pp. for the average F1-Score over two-stage learning and of 7.81 pp. over RUS. RUS achieved the worst results. Worth noting also is the difference in the average AUC between the tested methods. Two-stage learning achieved the same results as ROS and a 2.89 pp. higher score than RUS. Random minority oversampling is a technique that requires a lot of computer power due to the increased size of the training set. Therefore, it may not be feasible in all scenarios, especially for a hyperparameter search procedure. Two-stage learning, while still performing well, requires the model to be pre-trained on excess data only once and then a hyperparameter search can be performed using the thresholded dataset. Random majority undersampling requires less computer power, although because of voluntarily discarding data it achieves worse results in comparison.

### 3.5. Comparison to Other Recent Models

In Table 6 we compare the results of our experiments to other works. Goals and test sets used across the works make the results not directly comparable. Previous studies reported the results with different metrics, which made them difficult to compare with each other and our study.

## 4. Discussion

The authors presented a model trained for retinal disease screening in the ageing societies of developed countries. The best utilized architecture (ConvNextTiny) reached 80.46 ± 1.48 overall accuracy, with average 81.20 ± 2.26 sensitivity and 92.96 ± 0.55 specificity. It was reported that ophthalmic consultants detect retinal diseases with 89% sensitivity and 86% specificity when relying on eye fundus photographs [75]. The presented model had a lower sensitivity and higher specificity than ophthalmologists, however these benchmarks proved its potential clinical utility. An average AUC of 95.10 ± 0.36 classified our model as an acceptable screening method with excellent classification performance [76]. The utilized dataset potentially contributed little to the result. Most of the database consisted of poor-quality retinal images, often blurred or distorted, annotated by different experts according to various guidelines. This could lead to ambiguous interpretation for each class in the dataset. However, this diversity gave a better approximation of a real clinical setting. The results were more reliable and generalizable to the true screening process.

Despite a lack of certain comparability, the authors attempted to compare the AUC between the presented model and the most recent studies. Although AUC remains the most reliable measure of the learning algorithm’s performance [77], only three studies reported this benchmark [11,14,16]. The model outperformed all three models in GL and AMD classification. The AUC for DR was higher than presented by Han et al. [14] and Bulut et al. [11], but Li et al. [16] obtained a better result.

Our model not only presented an acceptable performance, but also was the first that truly approximates to the real-world environment. Based on one of the largest datasets, it made the received results less biased and more generalizable than in previously published papers. The authors merged multiple datasets from around the world into one cumulative dataset. This minimized bias from the single-image acquisition process, ethnicity, or limited cameras models. The created model could be used in multiple clinics, located in distant places and which could be using different equipment, without a need for additional fine-tuning or calibration. This approach was partially adopted by Chellaswamy et al. [12] and Han et al. [14]. Chellaswamy et al. [12] merged 5 publicly available datasets and extracted with an undescribed method a limited number of fundus images for each of the analyzed diseases. As a result, the final dataset was relatively small and potentially biased due to the unknown method of image selection. Han et al. [14] combined 6 publicly available with 2 private datasets and achieved a large and diverse ophthalmic image collection. However, there was the potential for an even more diverse large-scale database, as 94 public, open-access, fully downloadable ocular image collections were available at the time when Han Y et al. [14] conducted their research [15]. Han et al. [14] utilized 2 proprietary image collections, thus full transparency of the model’s development could not be guaranteed. Neither the composition nor the collection process of the datasets was described in this paper. Moreover, these 2 private databases were collected from the Chinese population, which did not increase the overall ethnical diversity.

The presented study is the first study that only utilizes multiple public data sets. This made the presented findings fully reproductible by the scientific community. Previous models trained on multiple datasets have always encompassed at least one proprietary image collection. While Han Y et al. [14] mixed public and private collections, Bulut et al. [11] and Li et al. [16] developed their models exclusively with private datasets.

To date, this has been first study to address the problem of class imbalance in a large-scale database of retinal fundus images. The ODIR dataset, the most frequently utilized retinal image collection in published multiclass models [12,13,15,17] has severe class imbalance problems [13]. It seems reasonable to assume that merging multiple different datasets into one large one could even potentiate this issue. Our model exhibited an AUC > 90 in all included classes, despite large discrepancies in the number of images. The highest sensitivity and specificity were received for AMD and GL. Significantly lower benchmarks were reported for normal eye fundus and DR. Normal and DR had the highest shares in the final dataset, significantly greater than AMD and GL. The potential explanation of these findings is that the vast majority of normal eye fundus and DR images come from the EyePACS dataset, which describes its images as real-world data that may include noise in both the images and labels, artifacts, under- and overexposure [61]. Therefore, robust classification for this data may have proven the most challenging. Yet these conditions and the extensive cross-validation process in the fine-tuning stage of the model’s development made the received results the most reliable among recently published models. Gour et al. [13] partially approached the difficulty of class imbalance in the ODIR dataset. Although Gour et al. [13] supported their research with an analysis of class-wise performance, the developed model still showed higher sensitivity and accuracy for diseases with the highest prevalence in the dataset [13]. The model correctly classified fundus images of healthy retinas and glaucoma but failed to recognize other classes such as diabetic retinopathy or AMD [13]. Only one study aimed to address the problem of class imbalance in a dataset of retinal images [15]. Khan et al. [15] with unknown selection criteria created a balanced training set for the VGG-19 architecture model and utilized only the ODIR database. It cannot be excluded that the extraction process was biased, e.g., by selecting images with the highest quality and aimed to achieve the highest possible model performance.

Aside from advances in computer science, the presented model brought some novelty into the medical field. This was the first model targeted at the most distressing retinal diseases in ageing societies. Excellent AUC values for GL, AMD and DR proved its potential screening utility. The overall accuracy of 80.46 ± 1.48 meets the performance requirements for routine screening tests in medicine [72]. This model has been the first to be trained on medically relevant diseases. Apart from cataracts, which are not a retinal disease [24], the authors did not include rare diseases or ones irrelevant to screening such as retinitis pigmentosa. The inclusion of multiple less prevalent diseases in previous research [11,13,14,15] potentially decreased the screening utility of those models. Creating a model with multiple various diseases may be a curious academic problem. However, due to limited data availability and the tedious process of its collection, the creation of a real-world deep learning model with real clinical application should be restricted only to the most prevalent and distressing diseases, such as GL, AMD, and DR.

The present study has multiple limitations. Firstly, the developed model lacks a class that would signify “other” conditions—elderly people could suffer multiple other retinal diseases than GL, AMD, DR, and diseases could overlap with each other. However, public datasets include a limited number of classes of retinal disorders. Due to the almost infinite possibilities of “other” diseases, the model was simplified to these three most distressing diseases. Furthermore, the model’s performance was not validated by ophthalmologists. It is still uncertain whether the presented performance is comparable to that from a healthcare professional. The authors could not assume that retinal images from different datasets had consistent image classification. Retinal classification guidelines vary between countries, and even partial assessment of the final dataset by an experienced physician could be beneficial. Finally, authors did not have access to some datasets, which limited the number of images utilized. This could influence the final performance of the developed model.

## 5. Conclusions

This work presents classification results for the most distressing and screening-relevant retinal diseases: diabetic retinopathy, glaucoma and age-related macular degeneration, on the basis of multiple publicly available datasets, without performing an evaluation of private datasets gathered in controlled environments. Availability of the data and clear selection criteria ensured reproducibility of the results. The achieved results classified the developed model as a useful screening method and the data utilized made it more reliable. Merging multiple datasets mitigated the data bias problem. A class imbalance problem, potentiated because of dataset merging, was addressed via transfer learning, loss function weighting and two-stage learning procedures. Such a model can enhance the efficiency and effectiveness of eye care providers. This research fills the gap in the literature on multiclass models and contributes to improving the diagnosis and treatment of retinal diseases.

## Figures and Tables

**Table 1:** Image augmentations used during model training with the probability of their application.

Name	Probability	Values
Rotate	0.8	[−90°, 90°]
Horizontal flip	0.5	-
Vertical flip	0.5	-
Random brightness contrast	0.5	Brightness limit: 0.1
		Contrast limit: 0.15
Cutout	0.5	Number of holes: 20 Maximum height of hole: 11 px
		Maximum width of hole: 11 px

**Table 2:** Summary of datasets used for pre-training, fine-tuning and ROS/RUS experiments.

Split	# of Normal	# of Glaucoma	# of AMD	# of Diabetic Retinopathy	Total # of Samples
Pre-training	82,626	0	0	30,592	113,218
Fine-tuning	3787	3787	632	3787	11,993
ROS/RUS	86,415	3787	632	34,379	125,211

**Table 3:** Summary and characteristics of the datasets.

Dataset	N	GL	AMD	DR	Camera Models	Annotators
ACRIMA [54]	309	396	0	0	Topcon → TRC → non-mydriatic	Two glaucoma experts with 8 years of experience
APTOS 2019 Blindness Detection Dataset [55]	3733	0	0	1857	Variety of cameras	Not available
Cataract [56]	300	101	0	100	Not available	Not available
DR HAGIS [57]	0	10	10	10	Topcon TRC-NW6s non-mydriatic, Topcon TRC-NW8 non-mydriatic or Canon CR DGi non-mydriatic	Expert grader
DR1, DR2 [58]	895	0	0	1118	Topcon → TRC-50X mydriatic	Respectively, three and two medical specialists
DRIVE [59]	33	0	0	5	Canon → CR5 → non-mydriatic 3CCD	Ophthalmology expert
Machine learn for glaucoma [60]	788	756	0	0	Not available	Not available
e-optha [47]	116	0	0	121	Not available	Ophthalmology experts
Kaggle: EyePACS [61]	65,343	0	0	23,359	Variety of cameras	A panel of medical specialists
BAIDU: iChallenge-AMD [62]	311	0	89	0	Not available	Not available
REFUGE [63]	360	40	0	0	Zeiss Visucam 500 non-mydriatic	Seven glaucoma specialists
Davis Grading of One and Concatenated Figures [64]	6561	0	0	3378	Nidek AFC-230 non-mydriatic	Specialist grader
Longitudinal diabetic retinopathy screening data [65]	0	0	0	1120	Topcon → TRC-NW65 non-mydriatic	Two graders
Messidor-2 [66]	1017	0	0	731	Topcon TRC NW6	Medical expert
ODIR-5K [19]	3098	312	280	1697	Various cameras such as Canon, Zeiss, Kowa	Trained human readers with Quality control management
LAG [67]	3147	1711	0	0	Not available	Glaucoma specialists
RIGA [62]	0	289	0	0	Topcon → TRC → 50DX mydriatic	Six experienced ophthalmologists
RIM-ONE DL [68]	313	172	0	0	Kowa WX 3D stereo non-mydriatic or Nidek AFzC-210 non-mydriatic with a Canon EOS 5D Mark II body	Three experts
ROC [69]	0	0	0	100	Topcon NW 100, NW 200, or Canon CR5-45NM	Retinal experts
STARE [70]	36	0	61	92	TOPCON TRV-50	Ophthalmology experts
ARIA [71]	61	0	23	59	Zeiss FF450+ mydriatic	Retinal expert
RFMID [72]	669	0	169	632	TOPCON 3D OCT-2000, Kowa VX-10alfa mydriatic and non-mydriatic two in one, and TOPCON TRC-NW300 non-mydriatic	Two ophthalmologists
TOTAL	86,415	3787	632	34,379		

N: Normal fundus image; GL: Glaucoma; AMD: Age-related macular degeneration; DR: Diabetic Retinopathy.

**Table 4:** Performance metrics for each model with standard deviation computed over the ten cross validation folds. Values in bold are the best results obtained.

Class	Metric	ResNet50	RegNetY3_2gf	ConvNextTiny
Normal	F1-Score	72.61 ± 1.86	72.15 ± 2.32	**72.97 ± 2.60**
	Sensitivity	73.75 ± 3.49	73.75 ± 6.64	**74.57 ± 3.94**
	Specificity	**86.46 ± 1.64**	85.99 ± 2.90	86.27 ± 1.76
	AUC	90.53 ± 0.76	90.19 ± 0.77	**90.64 ± 0.56**
	Accuracy	**82.50 ± 1.27**	82.17 ± 0.88	80.01 ± 1.10
Glaucoma	F1-Score	**95.22 ± 0.80**	94.42 ± 0.83	94.83 ± 0.96
	Sensitivity	**95.64 ± 1.02**	95.11 ± 1.09	95.54 ± 1.22
	Specificity	**97.57 ± 0.64**	97.06 ± 0.51	97.25 ± 0.81
	AUC	**99.44 ± 0.18**	99.30 ± 0.23	99.32 ± 0.17
	Accuracy	92.78 ± 0.41	96.92 ± 0.66	**97.20 ± 0.66**
AMD	F1-Score	81.78 ± 4.35	79.25 ± 4.21	**82.98 ± 3.50**
	Sensitivity	84.01 ± 8.13	83.23 ± 6.86	**84.02 ± 6.37**
	Specificity	98.82 ± 0.43	98.51 ± 0.47	**98.97 ± 0.49**
	AUC	**99.25 ± 0.34**	98.99 ± 0.51	98.79 ± 0.83
	Accuracy	97.91 ± 0.29	98.13 ± 0.31	**98.14 ± 0.31**
Diabetic Retinopathy	F1-Score	72.32 ± 1.28	71.60 ± 2.21	**72.96 ± 1.78**
	Sensitivity	70.56 ± 2.23	69.11 ± 6.67	**70.69 ± 3.33**
	Specificity	88.65 ± 1.65	89.09 ± 3.30	**89.36 ± 1.91**
	AUC	91.15 ± 0.65	90.97 ± 0.64	**91.65 ± 0.87**
	Accuracy	82.63 ± 1.09	**83.04 ± 0.94**	80.66 ± 1.27
Average	Accuracy	89.88 ± 7.53	**90.15 ± 7.43**	88.99 ± 8.74
	F1-Score	80.48 ± 1.51	79.36 ± 1.60	**80.93 ± 1.61**
	Sensitivity	80.99 ± 2.13	80.30 ± 2.03	**81.20 ± 2.26**
	Specificity	**92.88 ± 0.40**	92.66 ± 0.39	92.96 ± 0.55
	AUC	95.09 ± 0.39	94.87 ± 0.41	**95.10 ± 0.36**
Overall	Accuracy	79.76 ± 1.39	79.53 ± 1.07	**80.46 ± 1.48**

**Table 5:** Performance metrics for each resampling method with standard deviation computed over the ten cross validation folds. All experiments were performed using the ConvNextTiny architecture. Values in bold are the best results obtained.

Class	Metric	Two-Stage Learning (Our)	RUS	ROS
Normal	F1-Score	72.97 ± 2.60	63.52 ± 3.50	**73.84 ± 1.36**
	Sensitivity	74.57 ± 3.94	63.27 ± 8.69	**80.34 ± 5.50**
	Specificity	**86.27 ± 1.76**	83.75 ± 3.76	82.83 ± 3.79
	AUC	**90.64 ± 0.56**	85.59 ± 0.67	90.23 ± 0.87
Glaucoma	F1-Score	94.83 ± 0.96	92.36 ± 1.00	**95.34 ± 1.28**
	Sensitivity	**95.54 ± 1.22**	91.08 ± 3.03	92.45 ± 3.07
	Specificity	97.25 ± 0.81	97.17 ± 0.91	**99.33 ± 0.45**
	AUC	99.32 ± 0.17	98.69 ± 0.18	**99.46 ± 0.12**
AMD	F1-Score	82.98 ± 3.50	76.16 ± 3.84	**84.44 ± 2.55**
	Sensitivity	84.02 ± 6.37	**93.01 ± 3.90**	75.40 ± 4.18
	Specificity	98.97 ± 0.49	97.13 ± 0.72	**99.82 ± 0.28**
	AUC	98.79 ± 0.83	99.05 ± 0.39	**99.28 ± 0.27**
Diabetic Retinopathy	F1-Score	**72.96 ± 1.78**	63.23 ± 2.87	72.89 ± 1.59
	Sensitivity	**70.69 ± 3.33**	62.43 ± 7.74	70.05 ± 4.84
	Specificity	89.36 ± 1.91	84.09 ± 4.37	**89.82 ± 2.74**
	AUC	**91.65 ± 0.87**	85.52 ± 0.84	91.41 ± 0.71
Average	F1-Score	80.93 ± 1.61	73.82 ± 1.43	**81.63 ± 1.31**
	Sensitivity	**81.20 ± 2.26**	77.45 ± 1.29	79.56 ± 1.38
	Specificity	**92.96 ± 0.55**	90.54 ± 0.43	92.95 ± 0.46
	AUC	**95.10 ± 0.36**	92.21 ± 0.41	**95.10 ± 0.40**
Overall	Accuracy	80.46 ± 1.48	73.35 ± 1.26	**80.65 ± 1.30**
Technical	Runtime [s]	1196.4	370	37,194.9

**Table 6:** Summary of the results obtained by our model and in related works. The results are difficult to compare, because each study had different aims, questions to answer and used different test sets. Because we used the most diverse dataset our metrics were the most reliable in terms of developing a model applicable in clinical screening.

Paper	Class	F1-Score	Sensitivity	Specificity	AUC	Accuracy
Ours (ConvNextTiny)	Normal	72.97	74.57	86.27	90.64	80.01
	Glaucoma	94.83	95.54	97.25	99.32	97.20
	AMD	82.98	84.02	98.97	98.79	98.14
	Diabetic Retinopathy	72.96	70.69	89.36	91.65	80.66
Han et al. [14]	Normal	-	-	-	-	-
	Glaucoma	-	83.70	84.00	91.60	83.89
	AMD	-	77.61	78.75	86.70	78.37
	Diabetic Retinopathy	-	80.36	80.50	89.10	80.39
Bulut et al. [11]	Normal	-	-	-	-	-
	Glaucoma	-	-	-	81.10	-
	AMD	-	-	-	96.30	-
	Diabetic Retinopathy	-	-	-	87.10	-
Gour et al. [13]	Normal	-	77.00	21.00	-	40.00
	Glaucoma	-	40.00	60.00	-	54.00
	AMD	-	06.00	93.00	-	88.00
	Diabetic Retinopathy	-	05.00	94.00	-	89.00
Chellaswamy et al. [12]	Normal	96.39	95.99	91.27	-	95.00
	Glaucoma	96.43	94.95	96.32	-	96.00
	AMD	93.96	99.01	94.98	-	96.38
	Diabetic Retinopathy	-	-	-	-	-
Muthukannan et al. [17]	Normal	94.09	95.65	98.56	-	99.20
	Glaucoma	97.04	97.77	99.28	-	97.80
	AMD	95.49	94.98	99.01	-	98.40
	Diabetic Retinopathy	94.98	94.31	98.92	-	97.90
Khan et al. [15]	Normal	-	-	-	-	-
	Glaucoma	92.00	97.00	-	-	-
	AMD	88.00	92.00	-	-	-
	Diabetic Retinopathy	89.00	92.00	-	-	-
Li et al. [16]	Normal	-	94.50	95.70	98.90	-
	Glaucoma	-	80.40	93.40	95.30	-
	AMD	-	85.80	93.90	97.60	-
	Diabetic Retinopathy	-	80.40	89.70	95.00	-

## Data Availability

Utilized datasets were public, while preparing this manuscript. To access any dataset please refer to relevant citation for link or guideline. All data enquires should be mailed to martin.tabakow@pwr.edu.pl.
